# Numerical and experimental investigation of a lighthouse tip drainage cannula used in extracorporeal membrane oxygenation

**DOI:** 10.1111/aor.14421

**Published:** 2022-10-21

**Authors:** Francesco Fiusco, Federico Rorro, Lars Mikael Broman, Lisa Prahl Wittberg

**Affiliations:** ^1^ FLOW, Department of Engineering Mechanics KTH Stockholm Sweden; ^2^ ECMO Centre Karolinska Astrid Lindgren Children's Hospital, Karolinska University Hospital Stockholm Sweden; ^3^ Department of Physiology and Pharmacology Karolinska Institutet Stockholm Sweden

**Keywords:** CFD, drainage, ECMO, flow structures, jet in crossflow, non‐Newtonian, PIV

## Abstract

**Background:**

Extracorporeal membrane oxygenation is a life‐saving therapy used in case of acute respiratory/circulatory failure. Exposure of blood to non‐physiological surfaces and high shear stresses is related to hemolytic damage and platelet activation. A detailed knowledge of the fluid dynamics of the components under different scenarios is thus paramount to assess the thrombogenicity of the circuit.

**Methods:**

An investigation of the flow structures developing in a conventional lighthouse tip (single‐staged) drainage cannula was performed with cross‐validated computational fluid dynamics and particle image velocimetry. The aim was to quantify the variation in drainage performance and stress levels induced by different fluid models, hematocrit and vessel‐to‐cannula flow rate ratios.

**Results:**

The results showed that the 90° bends of the flow through the side holes created a recirculation zone inside the cannula which increased residence time. Flow structures resembling a jet in a crossflow were also observed. The use of different hematocrits did not significantly affect drainage performances. The most proximal set of holes drained the largest fraction of fluid. However, different flow rate ratios altered the flow rate drained through the tip. The use of 2D data led to a 50% underestimation of shear rate levels. In the drainage zone the non‐Newtonian behavior of blood was less relevant.

**Conclusions:**

The most proximal holes drained the largest amount of fluid. The flow features and distribution of flow rates among the holes showed little dependence on the hematocrit. The non‐Newtonian behavior of blood had a small influence on the dynamics of the flow.

## Graphical abstract

This study investigated with numerical and experimental techniques a glass model of a lighthouse tip drainage ECMO cannula across different flow rates and with different viscosity models. The results show that the most proximal holes drain the largest fraction of fluid and the dynamics are governed by a jet in crossflow type of phenomenon. The non‐Newtonian behavior of blood was less relevant in the drainage zone.

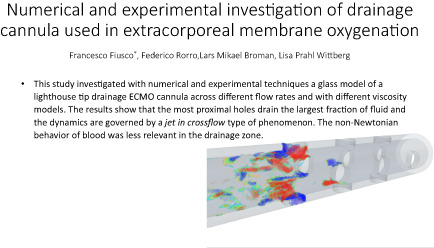

## 1 INTRODUCTION

Extracorporeal membrane oxygenation (ECMO) is a life‐saving therapy effectively used in the treatment of patients with severe respiratory failure, with or without heart failure. For extracorporeal gas exchange, vascular access is needed, and this is accomplished with one or more cannulae placed in major vessels.

The ECMO circuit is composed of a membrane oxygenator (artificial lung), a centrifugal/roller pump to drive the flow and tubing with connectors. However, blood is exposed to non‐physiological conditions where blood component damage/activation induce increased risks for hemolysis, thrombosis, embolism, and bleeding.^1^, ^2^ These adverse events are influenced differently in veno‐venous and veno‐arterial ECMO modes but with similar incidences.^3^ The most common causes of death during ECMO are intracranial bleeding or thromboembolic/ischemic complications.^4^, ^5^ In COVID‐19, ECMO has been reported to be associated with a 14% incidence of thromboembolic complications.^6^ Similarly, the seriousness of the high thromboembolic risk has been brought forward regarding ECMO treatment of children with COVID‐19.^7^ To mitigate the complication risk, ECMO requires adequate management of anticoagulation, artificial surfaces with biocompatible coating and direct intravenous administration of unfractionated heparin or direct thrombin inhibitors to the patient. Moreover, in the setting of blood pumps, blood damage and platelet activation are largely due to mechanical stresses induced by the non‐physiological flow field.^2^ Consequently, a thorough understanding of the fluid mechanics associated with the components is vital to assess the phenomena related to undesired hemodynamic effects.

Although a number of studies on cannula‐related adverse events have been performed,^8^, ^9^ relatively few studies have investigated cannula design and fluid mechanics as compared to e.g., the pumps.^10^, ^11^, ^12^, ^13^ ECMO cannulae are available in numerous designs and sizes ranging from 6 to 32 French (Fr, 1 Fr = 1/3 mm) in outer diameter and lengths from 6 to 80 cm.^14^, ^15^ Park et al. carried out a computational fluid dynamics (CFD) comparison of nine different cannulae focusing on the average wall shear stress and shear rates in relation to possible hemolytic damage.^16^ In a follow‐up comparison with different slanted side holes, the same group concluded that slanting may increase the flow rate (at the same pressure differential) while also decreasing the average shear rate in the cannula.^17^ Vatani et al. conducted a CFD study systematically varying cannula tip designs (side hole diameter, spacing and angle) in order to assess their influence on blood residence time and wall shear stress, concluding that a reduction in side hole diameter and spacing as hole slanting led to lower thrombogenic potential.^18^ The flow in a cannulated vessel gives rise to the development of some fundamental fluid dynamical patterns. In particular, the return cannula produces a flow field that resembles a *confined jet* where the ratio of vessel to cannula diameter sets the level of confinement. Although confined jets from an engineering perspective are well studied, few fluid‐dynamical studies addressed geometries as confined as found in ECMO cannulation.^19^ Regarding the drainage cannula, the flow is characterized by strong shear layers developing in the suction region.

The current study investigated a cannula in drainage configuration both numerically and experimentally. The aim was to gain knowledge on the basic fluid phenomena governing cannula flow and relate these findings to quantities of clinical interest. Thus, this study provides an assessment of the influence of Newtonian and non‐Newtonian viscosity, vessel flow to cannula drainage flow rate ratios and hematocrit (Hct) and their effect on the structures developing in the flow field. The implications in terms of drainage performance and shear stress levels were explored.

## 2 METHODS

**FIGURE 1:**
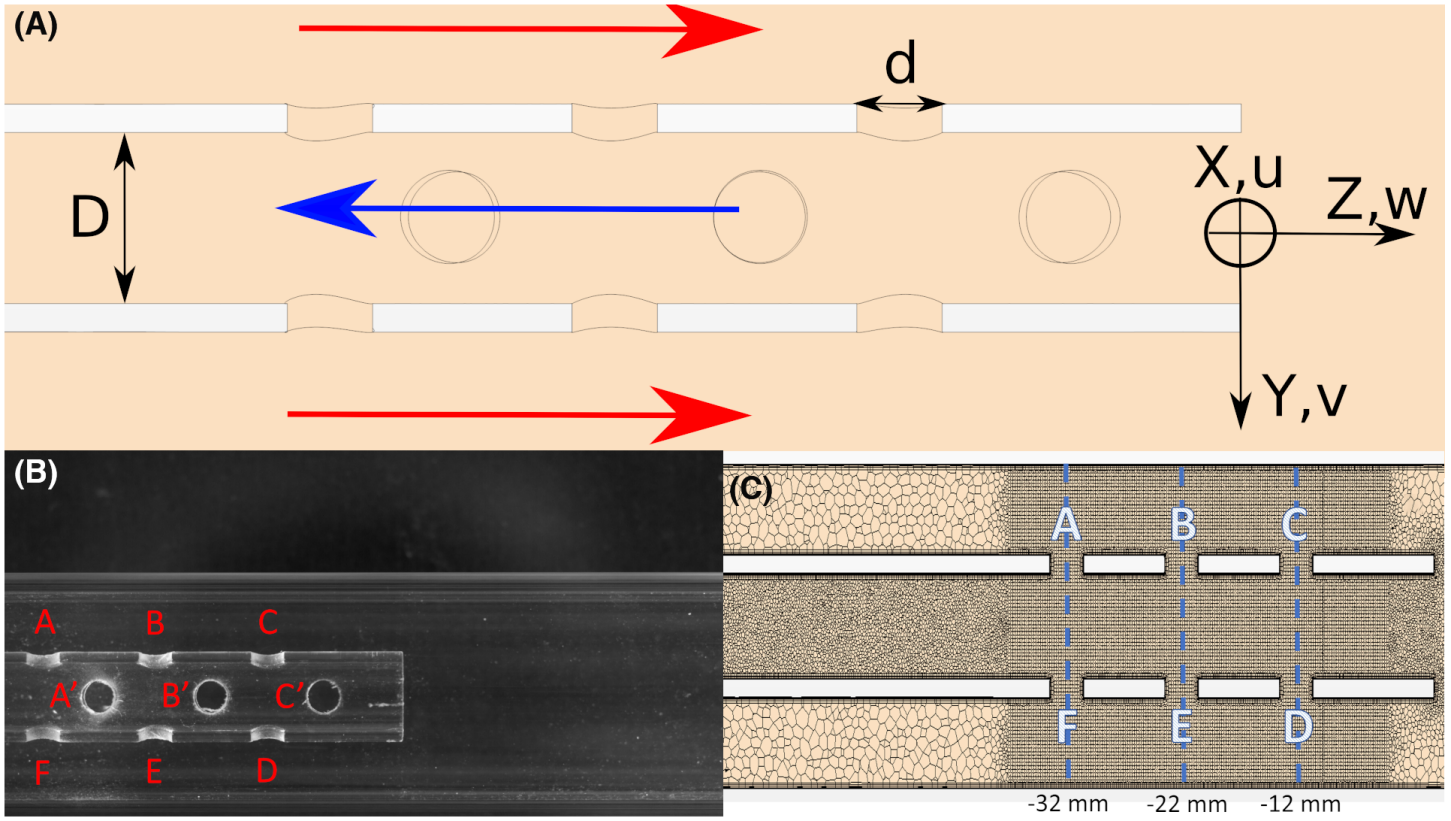
(A) A sketch of the domain with a focus on the drainage area. The red arrows show the direction of the vessel flow, whereas the blue arrow indicates the drainage direction. The origin of the coordinate system is placed on the centerline of the cannula at its tip. According to the coordinate system, *z* and *w* represent the position and velocity components in the streamwise direction. The inner cannula diameter is D = 6 mm and each side hole has a diameter d = 3 mm. (B) The cannula geometry used in the experiments and simulations. The labels A′, B′, C′ refer to the 90° shifted holes visible in the picture. The remaining holes on the opposite sides will be indicated as D′, E', F′. (C) Representation of the grid used for the simulations. A to F indicate six of the twelve side holes of the cannula, with A–F being the most proximal row.

This study investigated a lighthouse tip (single‐stage) ECMO cannula placed in a vessel to mimic a drainage configuration. The geometry is shown in Figure 1A, including the coordinate system used with (*x*, *y*, *z*) and (*u*, *v*, *w*) representing the position and velocity components, respectively. The *z*‐component is aligned in the streamwise direction, with the origin being placed on the centerline of the cannula tip. The external tube, the “vessel”, made of glass in the experimental setup, had an internal diameter of 18.3 mm. The cannula had a lighthouse tip with twelve side holes with a diameter of 3 mm placed in rows of three in each quadrant, as shown in Figure 1A. The cannula was a 24 Fr glass replica, i.e., the external diameter of the cannula was 8 mm, while the internal was 6 mm. The cannula length was 500 mm to ensure a fully developed flow in the drainage area. This cannula length is also representative of venous cannulae for clinical use.^15^

The investigated flow cases are shown in Table 1. Case 2 was the baseline numerical simulation, while Case 1 was its experimental counterpart used for validation. Cases 3, 4 and 5 were carried out to investigate the effect of different Hcts on flow features and draining performance, whereas Case 6 was set up to study a dynamically similar case to Case 2 but applying a non‐Newtonian blood viscosity model. Dynamic similarity was based on the Reynolds number, defined as:

(1) Re=ρUdμ

where *ρ* is the density, *d* is the considered inner cannula diameter, *U* is the bulk velocity and *μ* is the viscosity. The concept of dynamic similarity is based on the idea that a flow scenario can be described by a set of non‐dimensional numbers (in this case, the *Re* was used) and that different cases with the same *Re* will exhibit fundamentally similar physics; thus, by varying the quantities appearing in the Reynolds number so to keep it constant, similar behavior should be recovered. Cases 7, 8 and 9 investigated experimentally the effect of different flow rates on the draining characteristics.

**TABLE 1:** The investigated flow cases including the boundary conditions that were applied at the vessel inlet (vessel flow [positive] flow rate) and the cannula outlet (drainage flow rate, negative flow rate)

	Type	Vessel flow rate (L/min)	Drainage flow rate (L/min)	Fluid
1	Experimental	1.3	2.6	Water
2	Numerical	1.3	2.6	Water
3	Numerical	3	5	Blood Hct 30%
4	Numerical	3	5	Blood Hct 20%
5	Numerical	3	5	Blood Hct 35%
6	Numerical	3.64	7.28	Blood Hct 30%
7	Experimental	1.3	1.3	Water
8	Experimental	2.6	1.3	Water
9	Experimental	2.6	2.6	Water

*Note*: Cases 1 and 2 were used for cross‐validation, Cases 3 to 6 explored the effect of different hematocrits and scaling. Cases 7 to 9 were used to assess the effect of flow rate ratio on the drainage performance.

### 2.1 Numerical setup

The flow field was simulated applying a Large Eddy Simulation (LES) approach with no explicit sub‐grid scale model (implicit LES) and a fixed timestep of 0.0001 s. The 3D incompressible Navier–Stokes equations were discretized with formally second‐order accuracy and a second‐order implicit time integration scheme using the commercial CFD code Star‐CCM+ (v. 13.04.011 and 15.06.007). The cannula geometry was created using the Star‐CCM+ CAD client.

Regarding boundary conditions, a plug profile was prescribed at the inlet of the vessel (i.e., an annular positive flow, left to right, according to the coordinate system presented in Figure 1) and a fixed (negative) flow rate was applied at the cannula outlet to induce drainage flow. Applying a plug profile was justified by the location of the boundary condition (placed at *z* = −500 mm) allowing a fully developed flow to establish without affecting the flow region to be studied. The vessel boundary on the far right was placed at half a meter from the cannula tip to avoid boundary influence and treated as an inlet since all the simulated cases had higher drainage flow than vessel flow (see Table 1). All walls were treated with a *no‐slip* condition.

For the cases using water, a density of 997 kg/m^3^ and viscosity of 0.000887 Pa s were applied. For blood analog simulations, the Quemada model for blood viscosity was used^20^:

(2) $$\mu =\mu_{P}\;{\left[1-\frac{\alpha }{2}\;\frac{k_{0}+k_{\infty }\sqrt{\dot{\gamma }/{\dot{\gamma }}_{c}}}{1+k_{\infty }\sqrt{\dot{\gamma }/{\dot{\gamma }}_{c}}}\right]}^{-2}\;$$

where *μ*_*P*_ = 0.00132 Pa s is the plasma viscosity. The model coefficients *k*_0_, *k*_∞_, ${\dot{\gamma }}_{c}$ have been fitted to experiments in a range of shear rates with the following relationships^21^:

(3) $$k_{0}=\exp\left(3.874–10.41\alpha +13.8\alpha^{2}-6.738\alpha^{3}\right)$$

(4) $$k_{\infty }=\exp\left(1.3435–2.803\alpha +3.711\alpha^{2}-0.6479\alpha^{3}\right)$$

(5) $${\dot{\gamma }}_{c}=\exp\left(-6.1508+27.923\alpha -25.6\alpha^{2}+3.697\alpha^{3}\right)s^{-1}$$

where *α* is the hematocrit (as fraction) and $\dot{\gamma }$ is a scalar form of the shear rate (in this case, the infinity norm of the tensor).

Figure 1B shows the computational mesh used, consisting of 9 million cells. A mesh convergence study was performed by comparing time‐averaged velocity profiles at three locations (lines AF, BE, CD in Figure 1B). Three meshes of 4.3, 9 and 12.6 million cells were considered. The differences between the medium and fine meshes were negligible (Supporting Information S1). Also, the velocity gradients were well resolved: the Frobenius norm of the shear rate across the different meshes is provided in Supporting Information S1, showing a good agreement between the medium and fine meshes.

Each simulation was run for a physical time of 5.0 s and data was sampled with a frequency of 1000 Hz. All the presented data was obtained by ensemble averaging.

### 2.2 Experimental setup

The vessel‐cannula system was represented by two coaxial tubes, as shown in Figure 1A. Two circuits were used: one to drive the vessel flow in the vessel and another for the drainage by the cannula using a centrifugal blood pump. The setup was similar to that used by Lemétayer et al.^19^ The velocity field measurements were acquired using Particle Image Velocimetry (PIV). Zirconium dioxide particles with a diameter of 5 μm were used as tracers and the system comprised a high‐speed Nd:YAG laser (LPY703 50‐200, Litron Laser, Rugby, England) and a Dantec highspeed double frame CCD camera (Speedsense M120, Dantec Dynamics, Skovlunde, Denmark) with a 105 mm Nikkor lens (Nikkor, Nikkon, Tokyo, Japan). A laser sheet with a thickness of 1 mm was created with a system of lenses and aligned through the cannula centerline. A total of 1540 image pairs were acquired for each case at a frequency of 200 Hz, with a magnification factor of 27.5 pix/mm. Since PIV was conducted on a wall‐bounded, static geometry, the edges of the cannula and of the vessel were removed by each image before the cross‐correlation step took place. This was achieved by subtracting the mean of the image series from each frame, removing the light reflected by the glass and only retaining the light reflected by the particles in the flow. The velocity fields were generated from the PIV measurements using the software DynamicStudio v. 2015a (Dantec Dynamics) for an interrogation window that went from 35 mm upstream of the cannula tip to 35 mm downstream. In this configuration, the number of spurious vectors never exceeded 1%, with a total vector count in the domain of 238 × 63. Consistency of PIV measurements was assessed by computing the flow rate at the inlet and outlets of the domain, confirming that the obtained velocities satisfy the continuity equation. Time‐averaged velocity fields for all the experimental cases are shown in Supporting Information S2.

## 3 RESULTS

### 3.1 Experimental versus numerical simulations: Validation and verification

**FIGURE 2:**
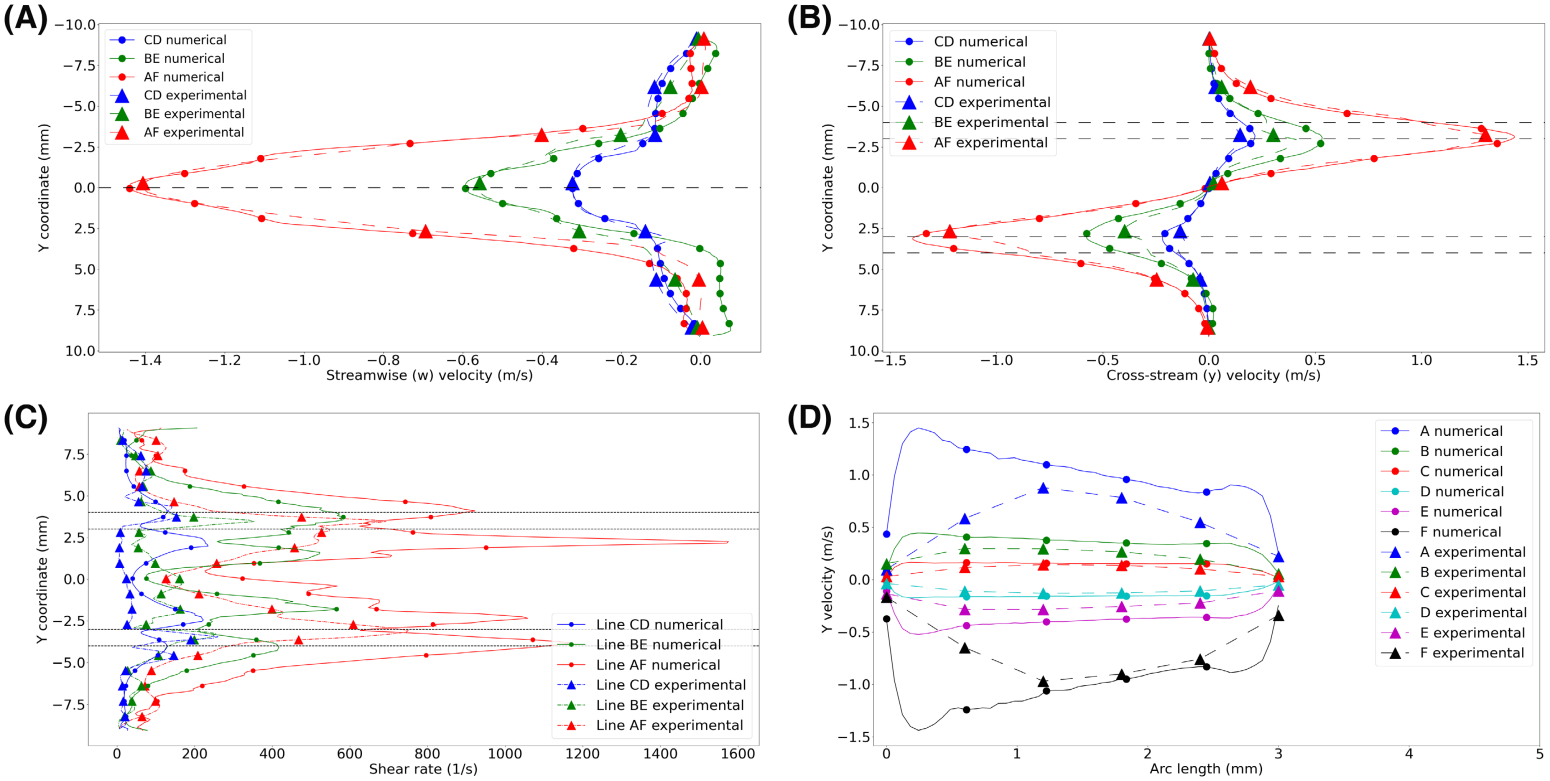
Comparison between numerical and experimental results. For the profiles taken at the centerline of the holes AF (*z* = −12 mm), BE (*z* = −22 mm), CD (*z* = −32 mm), (A) shows the streamwise (*z*) velocity, (B) shows the normal (*y*) velocity, (C) the shear rate norm. (D) shows the y velocity profiles taken across the side holes diameters (in the *z* direction). In panels B and C, the black dashed lines indicate the position of the cannula walls.

Comparing Cases 1 and 2, Figure 2A,B show a good agreement between experimental and numerical results for the streamwise (*w*) and cross‐plane (*v*) velocity profiles sampled on the centerlines of the hole rows (AF, BE, CD) inside the cannula. The agreement for line BE was less pronounced (also regarding the velocity gradients across the different meshes), as this region was characterized by highly unsteady flow. However, the flow structures developing inside the cannula were considered well captured numerically and experimentally. Not only is the flow inside the cannula of interest, but also the ability to capture the flow entering the cannula through the side holes. Figure 2D shows the *v* velocity profiles across the side holes for Cases 1 and 2, considering the six holes named according to Figure 1B. For the flow through the side holes, clear differences in velocity profiles were revealed. This was due to wall effects (reflections and diffraction) affecting the ability of the PIV measurements to capture the flow in this region, an aspect further addressed in the Discussion section.

Figure 2C shows the Frobenius norm of the shear rates sampled on lines AF, BE, CD. For the experimental case, it was computed using the two‐by‐two tensor of the *y* and *z* component derivatives, i.e.:

(6) $$\dot{\gamma }=\left[\begin{array}{cc} \frac{\partial v}{\partial y} & \frac{1}{2}\left(\frac{\partial v}{\partial z}+\frac{\partial w}{\partial y}\right)\\ \frac{1}{2}\left(\frac{\partial v}{\partial z}+\frac{\partial w}{\partial y}\right) & \frac{\partial w}{\partial z} \end{array}\right]$$

whereas for the numerical case the full shear rate tensor was used. Especially in regions of high shear, the 2D experimental data was found to differ from the numerical 3D data by almost 50% when comparing the total shear rate.

### 3.2 Drainage flow dynamics

**FIGURE 3:**
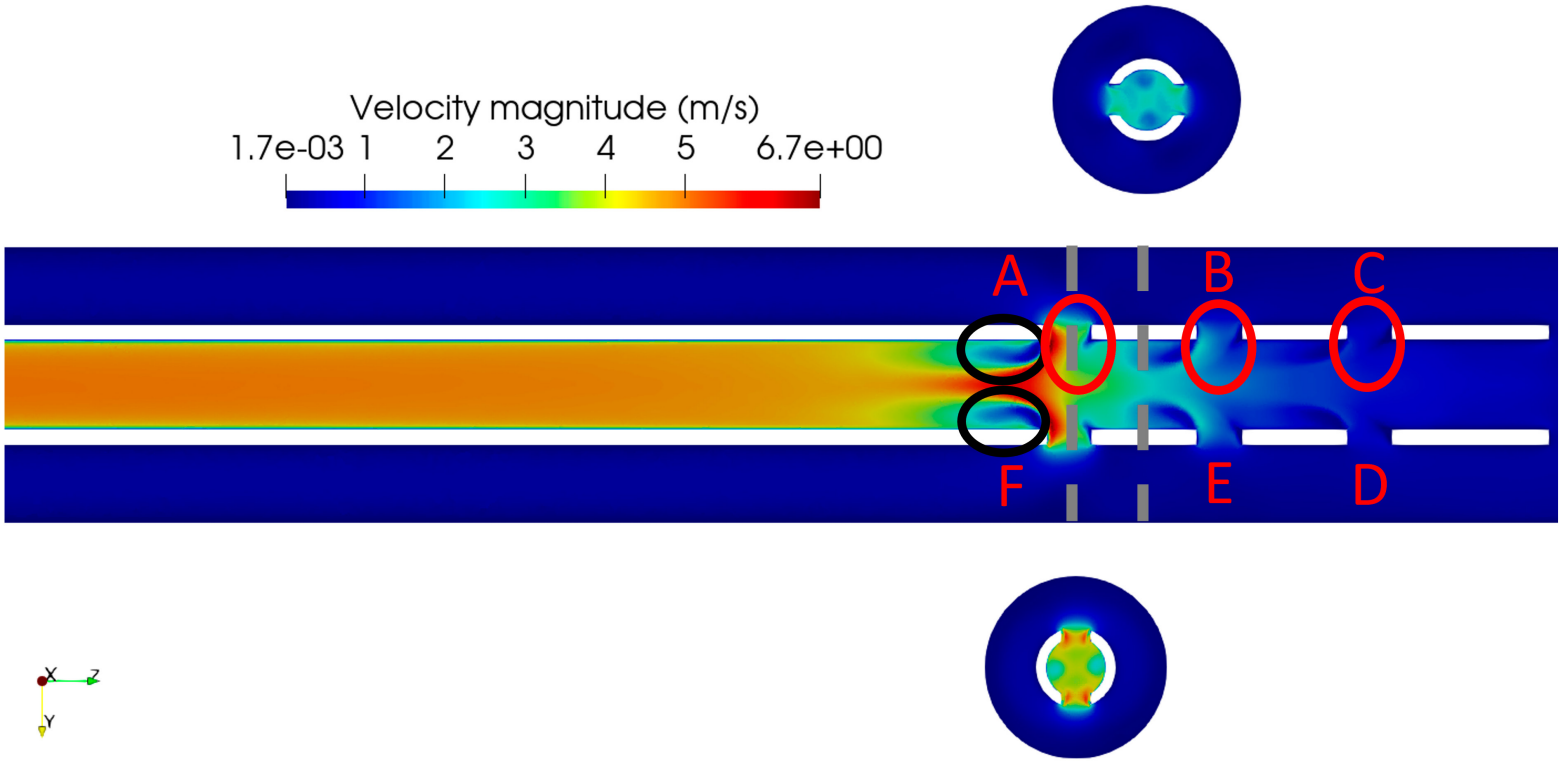
Averaged velocity field of Case 6 in the mid *zy* plane. The gray dashed lines show the location of the cross planes. The red circles highlight the jets in tandem, with the crossflow being the flow coming from the right side of the domain. The black ovals show the locations of the recirculation bubbles developing behind the 90° bend of the side holes.

The flow field for Case 2 (the validation case) is shown in Supporting Information S3. The velocity field was characterized by unsteadiness and fluctuating velocities (and hence stresses) both in space and time, with a swirling motion developing behind the most proximal holes (A–F, placed at the furthest from the tip) in the drainage direction. These flow features are highlighted in Figure 3, representing the averaged velocity field.

**FIGURE 4:**
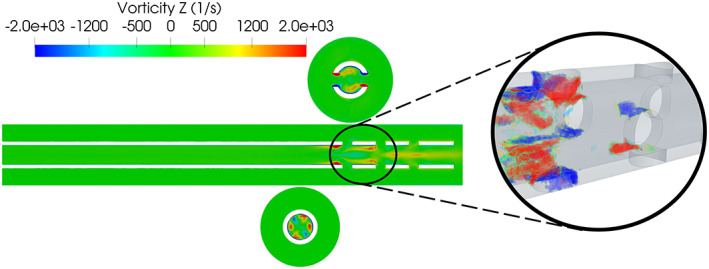
The figure shows the axial vorticity in the *zy* plane. The counter‐rotating vortex pairs are visible in the cross‐planes. The zoom‐in depict regions of high magnitude of helicity in the vicinity of the most proximal holes, with the red and blue clusters representing high positive and negative helicity respectively.

A dominant feature was the entrainment of the fluid from the vessel into the cannula leading to a more than 90° bend of the flow with respect to the axial direction, creating large shear stresses and small‐scale fluctuations. Especially for the proximal holes, A–F, this resulted in the development of a strong recirculation bubble behind the hole, marked by the black circles in Figure 3 (clearly visible in S3). Recirculation bubbles are undesirable from a hemodynamic perspective, inducing long residence times and stresses that may lead to increased risk of clot formation in the region of the side holes. The entrained flow emerging from each side hole met the drained stream and created a pattern closely resembling that of a jet in a crossflow.^22^ In this case, a *tandem* of jets in crossflow was present. A characteristic feature of jets in crossflows is the development of a counter‐rotating vortex pair in the axial direction,^23^ shown here in terms of axial vorticity in Figure 4. Figure 4 also shows that the bending of the jets was strong enough to minimize interaction between the two opposing jets coming from holes A and F inside the cannula, as shown in the cross‐planes; the same was observed for side hole pairs B–E and C–D. This interaction induced a swirling motion depicted in Figure 4 (see Video S3). Considering the individual terms of the vorticity equation, the evolution of vorticity inside the cannula was found to be dominated by convective transport and secondarily by vortex stretching, especially around the A–F holes (vorticity dissipation being around 1% of transport and stretching in that area).

The A–F holes shielded the downstream holes (B–E, C–D respectively), both in terms of developed vorticity and magnitude of the velocity, shown in the zoom‐in of Figure 4 by regions of high helicity (*H* = **u**·*ω*, where **u** is the velocity and *ω* is the vorticity).

**FIGURE 5:**
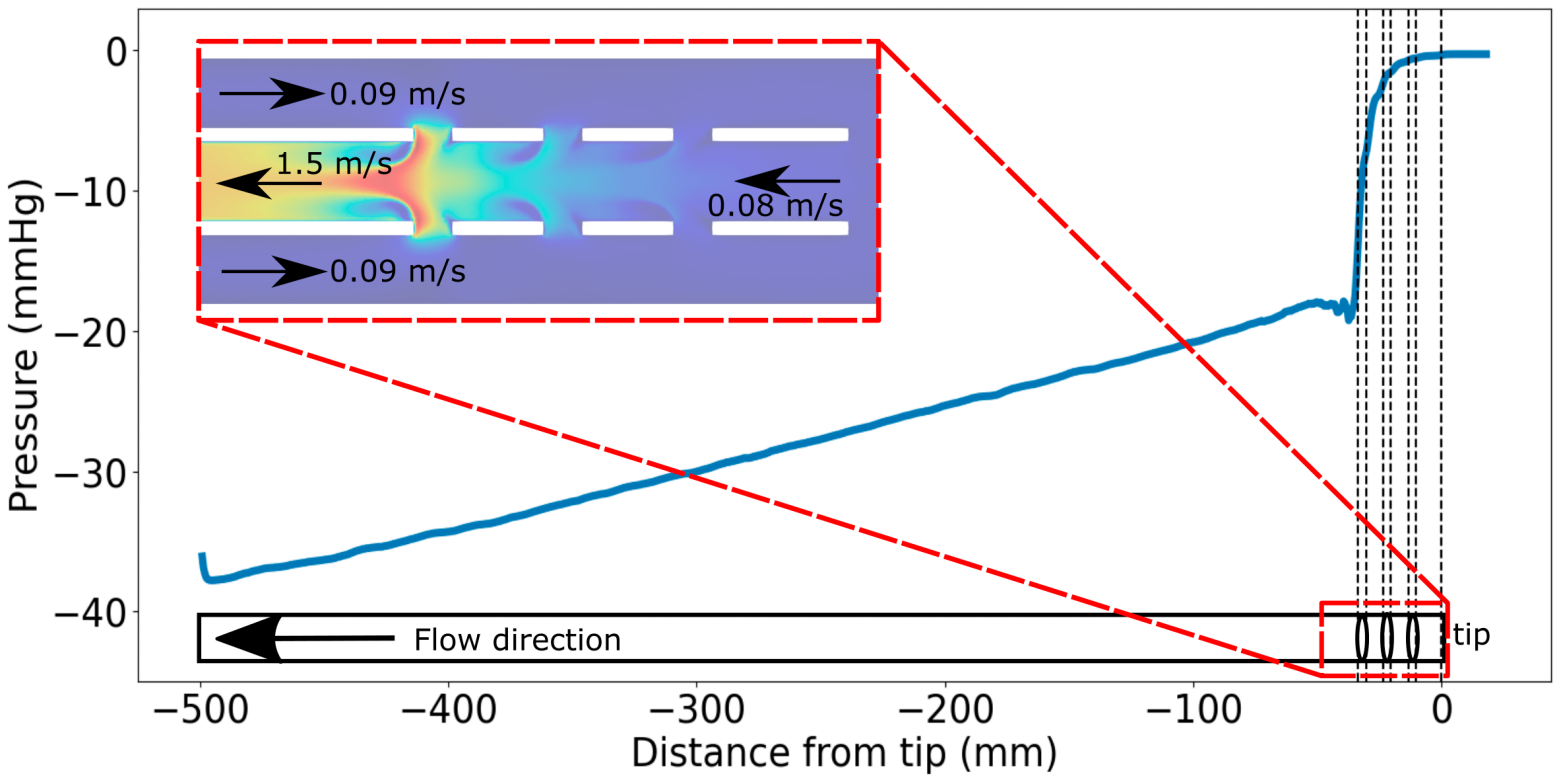
Pressure gradient along the centerline of the cannula for Case 2. The black dashed lines show the location of the side holes and the cannula tip. The zoom‐in of the drainage area is colored by velocity magnitude. The arrows in the panel show the bulk velocities of the drainage flow and vessel flow computed by diving the flow rates by the respective cross‐sectional areas.

The lower velocities emerging from the B–E, C–D holes (Figure 2D) were due to two phenomena:

- The drainage flow rate was larger than the vessel flow rate. As shown in the zoom‐in of the drainage area in Figure 5, this resulted in larger negative velocity in the cannula compared to the vessel flow. Therefore, at the first point of contact between the two flows (holes A and F) a strong suction was induced. Moving towards the tip, the velocity decreased, resulting in a lower fraction of fluid being drained by the distal holes.
  This led to a positive pressure gradient (shown in Figure 5, where *p*(*x*) is the gauge pressure with respect to the zero‐level) being developed inside the cannula (decreasing velocity along the *z*‐direction), which became less steep across the distal holes.
- The holes furthest from the tip (A–F) had a shielding effect, forming a “plug”, preventing the flow being drained from the vessel in the downstream holes (B–E, C–D). As shown in the video in Supporting Information S3 (and experimentally in Supporting Information S2), a cluster of high streamwise velocity was present outside of the cannula in the corner of holes A and F, limiting the amount of fluid available to be drained through holes B–E and C–D.

**FIGURE 6:**
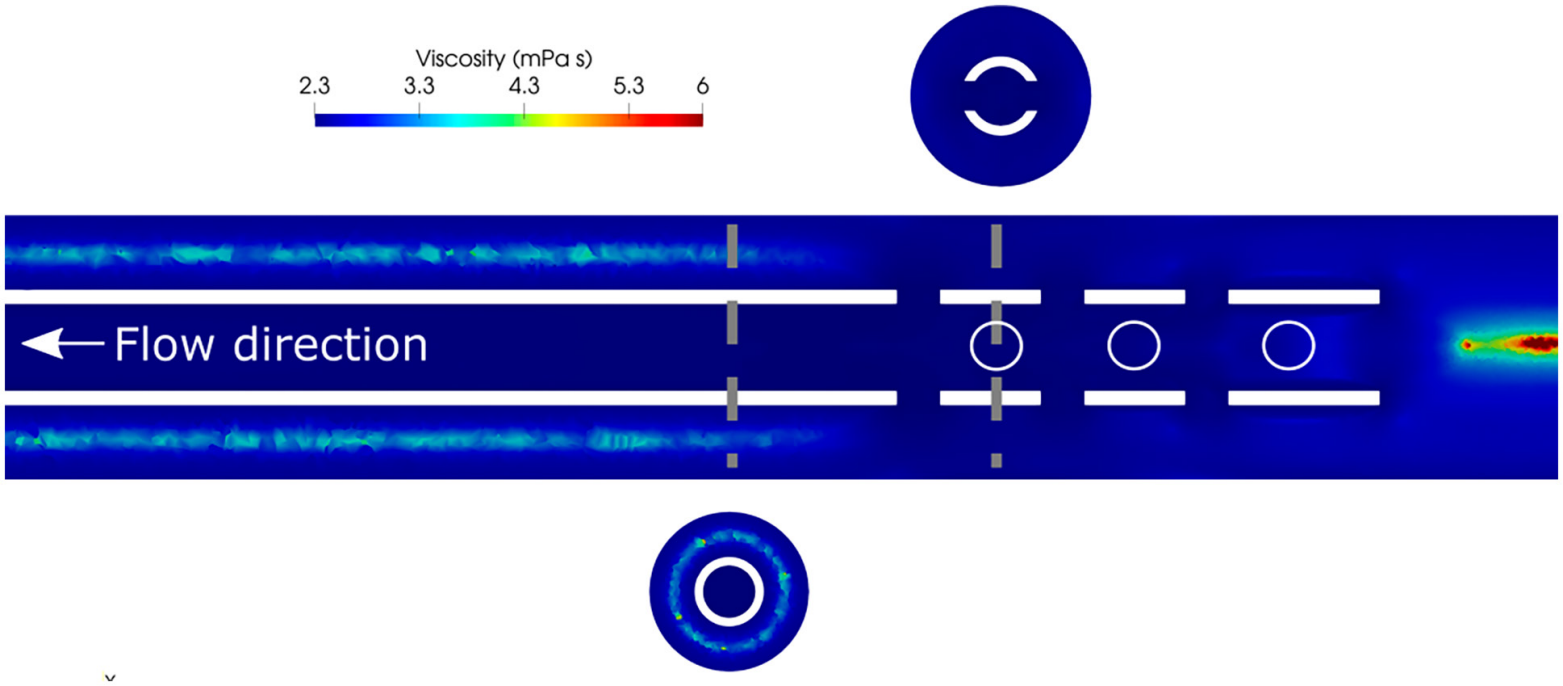
Viscosity field for Case 6. The gray dashed lines mark the location of the cross planes. Dark blue areas depict the locations where viscosity reached its asymptotic value, while dark red areas are more influenced by non‐Newtonian effects.

Case 6 was designed based on Case 1 using a fixed Reynolds number. The *Re* numbers of the flow for Case 1 was computed using the size of the cannula as the reference length, the bulk velocity of the drainage flow inside the cannula and the viscosity of water, whereas the *Re* of the vessel flow was computed considering the difference between the diameters and the bulk velocity of the vessel flow(Revessel_flow=dvessel−DUco−flowν). For Cases 1 and 6, *Re* was 12 000 for the drainage flow. The velocities were thereafter scaled using the asymptotic viscosity of blood with Hct 30 to quantify the importance of non‐Newtonian effects. The viscosity field for Case 6 is depicted in Figure 6. In most of the domain, the flow was largely dominated by strong shear layers and vortical structures, especially inside the cannula, inducing large shear rates. Zones of higher viscosity (i.e. lower shear) were present in the bulk of the vessel flow, both in the cannulated region and upstream of the cannula. Some cells in the cannulated area reached twice the asymptotic value, while far away from the cannula peaks of 0.0081 Pa s (three‐fold the asymptotic value) were observed. However, the areas were confined in space and did not interact with the drained flow, suggesting that in this configuration a Newtonian approximation can be used to describe fluid behavior in the holed region of the cannula.

### 3.3 Drainage performances

One item of clinical interest is the detailed drainage performance, i.e., the distribution of the flow rate among the different holes, shown in Table 2. For the experimental cases, flow rates were computed from the *v* velocity profiles across the side holes, using an axisymmetric approximation. However, according to the numerical data, the velocity displayed an asymmetric profile in the *y*‐component across holes A–F (Figure 2D). Two effects play a role in the comparison between flow rates computed from numerical or experimental data: (a) the shape of the velocity profile, which in the simulations has a peak close to the wall, whereas in the experimental case peaks in the centerline; (b) the fact that using a 2D velocity profile to compute the flow rate implies assuming that the jet has an axisymmetric shape at the level of the side hole, which deviated from the numerical data. Consequently, care should be exercised in the use of 2D data, due to the strongly three‐dimensional flow behavior.

**TABLE 2:** Fractional flow rate distribution for all investigated cases

Case	A	F	A′	F′	B	E	B′	E′	C	D	C′	D′	End hole
	%	%	%	%	%	%	%	%	%	%	%	%	%
1	11.6	13.0			4.48	4.29			2.03	1.86			11.5
2	15.0	15.1	8.92	8.81	5.42	5.13	3.27	3.18	2.15	2.09	1.3	1.27	11.5
3 (num)	14.9	14.8	9.11	8.92	5.86	5.87	3.07	3.05	2.34	2.32	1.18	1.15	6.81
4 (num)	15.0	15.0	9.25	9.10	5.52	5.53	3.16	3.12	2.19	2.18	1.15	1.20	7.40
5 (num)	15.0	14.9	9.14	9.00	5.66	5.68	3.28	3.17	2.27	2.28	1.21	1.29	6.29
6 (num)	15.0	15.0	8.57	8.53	5.21	5.24	3.21	3.17	2.24	2.24	1.30	1.29	10.9
7 (exp)	14.8	15.0			6.10	5.82			2.48	2.49			2.73
8 (exp)	17.3	17.3			6.60	6.55			0.22	1.18			0.05
9 (exp)	13.0	14.0			5.10	5.40			2.21	2.14			1.40
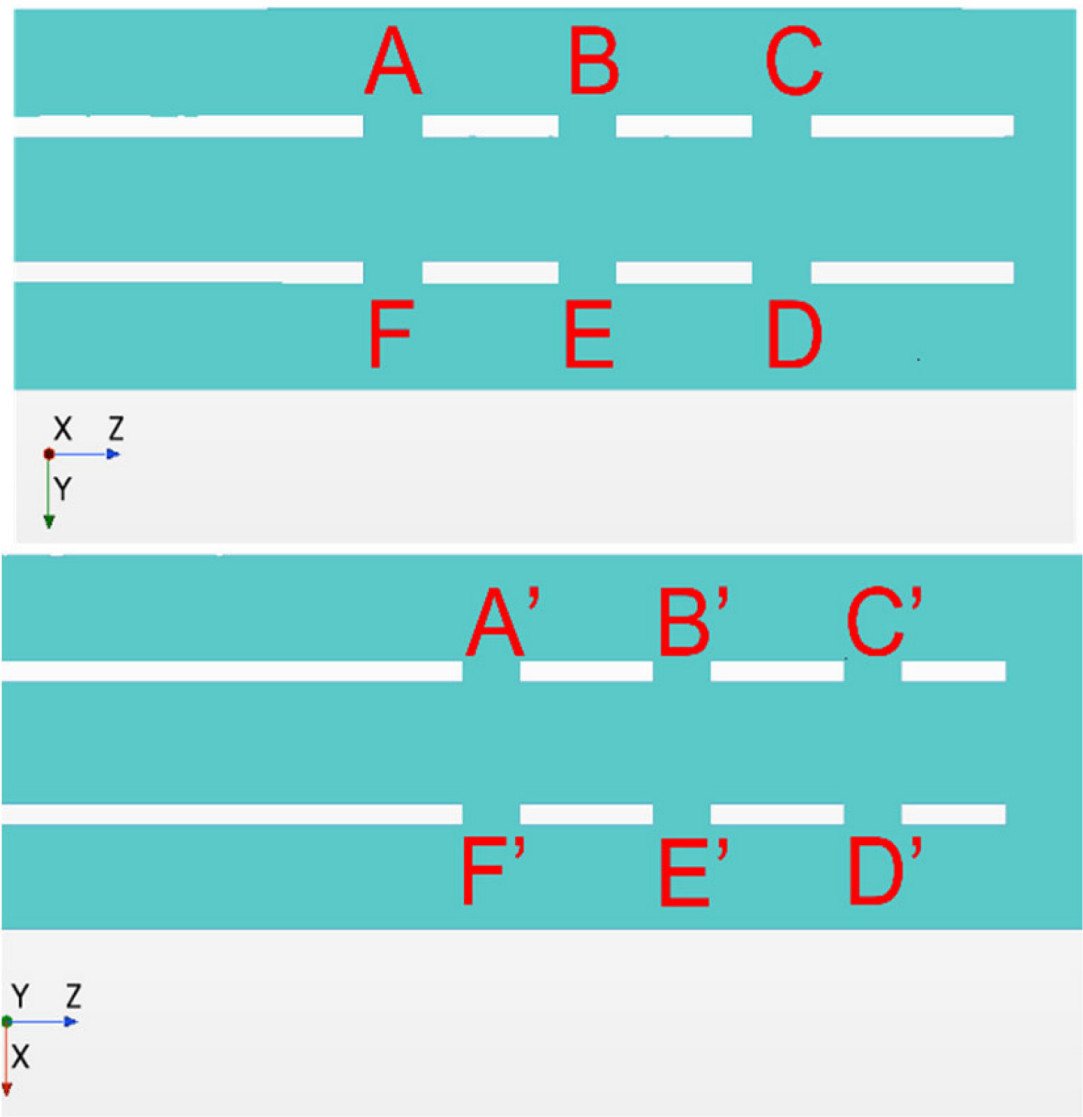													

*Note*: The bottom panel shows the location of the out‐of‐plane side holes.

Abbreviations: exp, experimental; num, numerical.

Regarding the role of viscosity, there is no appreciable effect of different Hcts on the distribution of flow across the holes: Cases 3 to 5 showed similar fractions. Notwithstanding the small difference in ratio between drainage flow and vessel flow compared to the validation case (0.5 for water case and 0.6 for blood), the draining performance resemble that of Case 1. Case 6, a Reynolds‐scaled Case 2, showed a similar flow distribution as the latter. The high shear rates result in a close to constant viscosity also using a non‐Newtonian fluid model. Thus, the global properties of this flow could be recovered using water by adequately adapting the boundary conditions. The flow drained by the out‐of‐plane holes was consistent across the different cases and with the trend moving from the proximal to the distal holes, suggesting that there is little interaction between the 90° shifted holes: the fraction of drained flow correlated to the distance from the cannula outlet (Table 2).

As for the experimental flow rate ratio investigation, no major differences were observed in the side hole distributions, apart from Case 8 (vessel flow double the drainage flow). However, the effect on the fraction of flow rate drained by the tip hole was more pronounced. For Case 8 even more flow was drained through holes A–F at the expense of holes C–D. However, the end‐hole drained a significantly lower fraction as compared to the other scenarios. Since the vessel flow was the same as the drainage flow for this case, a smaller amount of fluid was entrained from the far right of the domain compared to the other cases, thus explaining the lower flow rate through the tip hole. In all the cases, the set of holes furthest from the tip (A–F) drained the most. In terms of average shear rates, Cases 2 and 9 had the highest average (∼65 s^−1^, see Supporting Information S4), with also a low fraction of cells of the domain reaching values as high as 1750 s^−1^; Case 7 exhibited the lowest peaks and average shear rates, with a mean value of 37 s^−1^. The distributions are shown in the Figure S4.

**FIGURE 7:**
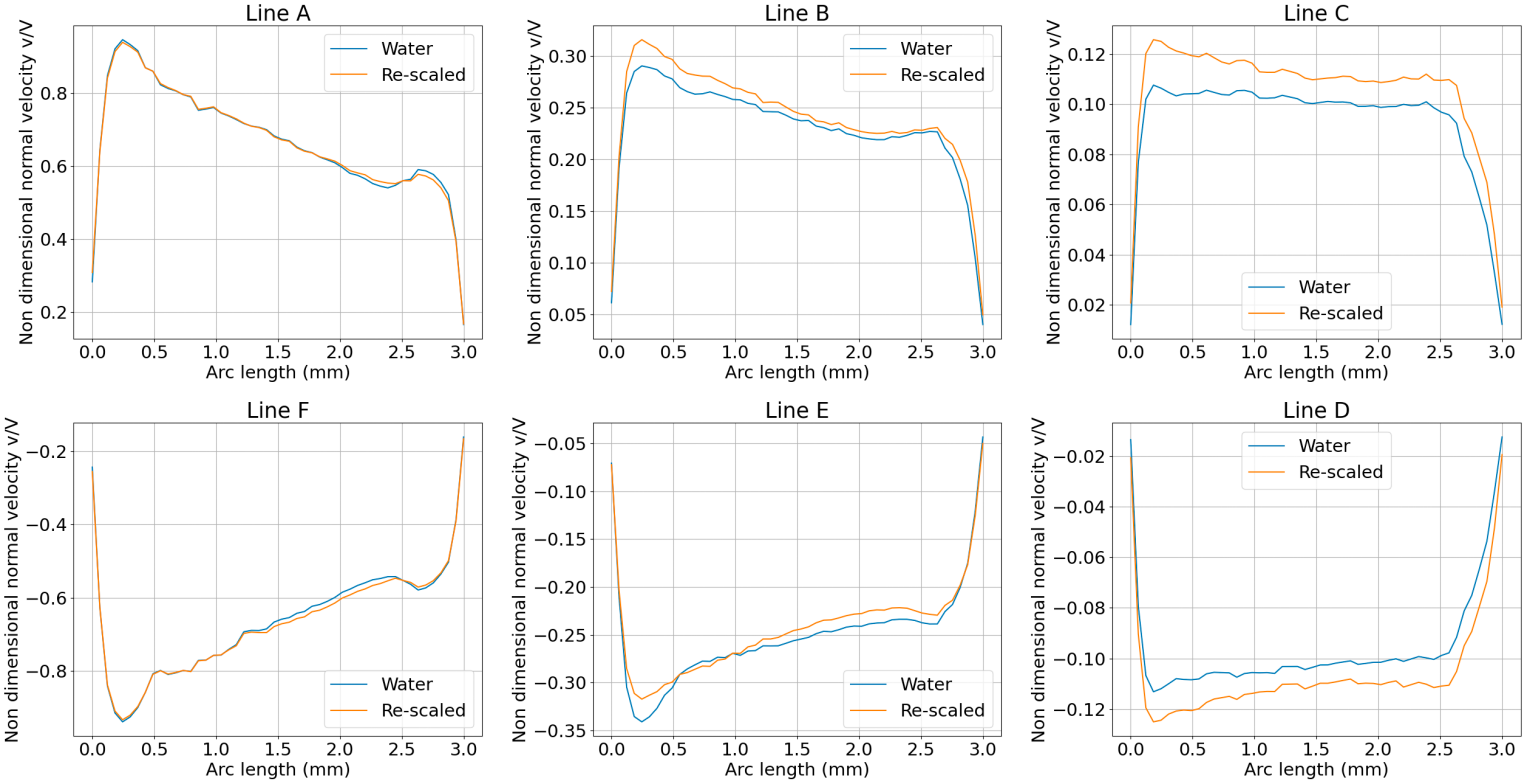
Normal (*y*) velocity profiles along each side hole diameter (*z* direction) for Cases 1 and 6, scaled using the bulk velocity of the drainage flow.

Due to the flow resemblance in Case 6 versus Case 2, the normal velocity profiles to the side holes for Cases 2 and 6 were scaled with the bulk drainage velocity (i.e., drainage flow rate divided by cross‐sectional area of the cannula). The curves in Figure 7 showed good agreement, implying that this scaling factor is sufficient to capture the inflow profile at the side hole using water experiments.

## 4 DISCUSSION

A model of a lighthouse tip ECMO cannula was investigated using both numerical and experimental techniques. The results showed that the flow field was dominated by structures similar to a *jet in crossflow*. These were triggered by the suction through the side holes. The proximal set of holes drained the largest amount of fluid across all the investigated cases, regardless of flow rate ratio between drainage flow and cannula flow and used fluid model, as earlier reported by Lindholm.^24^ Due to the convective transport dominating over viscous diffusion, the non‐Newtonian behavior of blood had less impact on the observed flow structures for the considered Hcts. This allowed to recover the drainage properties when using water by appropriately scaling the boundary conditions to keep the *Re* number constant.

Experimentally, two areas of uncertainty were observed: (1) The presence of a strong 90° bend reduced the number of particles that travel through the hole, thus inducing a smaller resolution of the field, (2) Particles in regions near the cannula boundaries were shadowed by the higher brightness of the cannula wall due to laser sheet reflection and absorption. As the mean of the image series was calculated and subtracted from the acquired images, those regions had much lower density of tracer particles than other parts of the domain when the PIV algorithm was applied. In particular, the numerical results showed that the velocity field through the side holes (especially for A–F) was strongly asymmetrical, a feature not reflected by the experimental results. For a similar scenario, the results by Vatani et al. showed close resemblance, with an asymmetric streamwise velocity profile developing across the most proximal holes' diameters.^18^ The combined effect of reflections, lower number of particles and overall lower resolution impacted the results. Thus, the computation of the flow rate through the holes using an axisymmetric approximation should be exercised with care to account for this effect. However, the streamwise velocity profiles across the centerlines of the holes (as in Figure 2A) suffered less from the influence of hole effects, showing a better agreement between simulations and experiments.

For all cases investigated, the flow was inherently unsteady, characterized by strong 3D effects such as a swirling motion behind the most proximal holes. The dominant flow feature was the fluid entrainment through the side holes, resulting in a *jet in crossflow* type of configuration.^22^ In the flow case studied, multiple jets were organized in a tandem, with the most proximal set providing a shielding effect on the other jets, which experienced lower crossflow velocities (and vorticities). This is consistent with previous results in literature.^25^ However, in our work, the crossflow was not uniform, making this flow scenario deviate from the ideal canonical case. Moreover, the origin of the jets, being a suction phenomenon rather than an injection, resulted in the jet not being axisymmetric at the level of the side hole (as it is commonly assumed in the canonical jet in crossflow). Instead, the jet velocities were higher towards the proximal side of the hole. However, some characteristics of the canonical jet in crossflow were retained. For example, the shear layer between the jet and the crossflow had a periodic “shedding” due to vortex roll‐up.^23^ For the most distal set of holes (where the conditions were the closest to the canonical configuration), the frequency was consistent with literature (corresponding to *f* = 120 Hz for a water case, leading to a Strouhal number of 1.2 using the crossflow velocity). For a similar jet‐to‐crossflow velocity ratio, *St* = 1.14 was reported.^23^, ^26^ The average jet‐to‐crossflow velocity ratio varied from about 2 to 1 moving from A–F to C–D for the numerical cases. This resulted in increasing bending of the jet after entering the cannula. Even when the vessel flow was higher than the drainage flow, the flow distribution did not change, suggesting that the drainage performances related to side holes are rather insensitive to the flow rate ratio. A strong pressure gradient developed from the outlet of the cannula to the most proximal set of holes due to cannula length, explaining the reason for the most proximal holes draining a larger quantity of fluid: the pressure gradient between different hole rows was not of comparable magnitude, thus decreasing the jet momentum.

Moreover, behind the jets, a strong recirculation bubble developed where high shear stress due to the presence of wake vortices and long residence times are expected. Thus, the 90° bend represents a critical area of the domain concerning activation properties as shown in the case of a left‐ventricular cannula.^27^ Especially at lower flow rates, prolonged residence time is a promoter of clotting; the recirculation zones behind the proximal holes could thus induce such phenomena. Levels of shear rate as high as 50% of the magnitude at holes A–F were reached in holes B–E and (albeit much lower) C–D. However, those holes only accounted for <15% of the total drained flow. Such high stresses could be damped by slanted side holes, as suggested by Park et al.^17^

The viscosity field in Figure 6 showed that for this flow scenario, the assumption of treating blood as a Newtonian fluid represented by an asymptotic viscosity dependent on the Hct is adequate. However, the difference induced by the three clinically relevant Hcts that were considered did not result in any significant flow effects. Hence, smaller variations in Hct (in the order of 15%) were not found to affect the flow structures and draining performance to a notable extent. The same was observed for the shear levels. Nevertheless, it should be noted that the used viscosity model assumed a constant (space‐invariant) Hct. Applying simulations to predict hemolysis (or thrombus formation), the effect of red blood cell transport on the viscosity should be considered.

Variations in boundary conditions (vessel flow to cannula flow rate ratio) had a strong impact on cannula behavior, although the amount of fluid drained through the side holes was less sensitive to the changes within the parameter ranges considered in this work. In particular, the drainage flow rate impacted the shear rates, inducing peaks twice the other cases (comparing Cases 2 and 9 with 7 and 8). It should also be noted that changing flow rate ratio considerably influenced the amount of fluid drained from the end hole. This was a direct consequence from the fact that, with a larger flow rate coming from the inlet of the vessel, a lower amount of fluid had to be entrained from far away from the cannula (larger *z* coordinate).

While the levels of shear may not be relevant for hemolytic damage, platelets trapped in such vortical motions are likely to undergo important levels of stress. The computation of the flow rates through the A‐F holes should consider the effect of non‐asymmetry of the flow, less apparent from the experimental data. Nevertheless, given that blood behaves like a Newtonian fluid in the vicinity of the holes, a simple Reynolds number analogy is effective in recovering global properties like flow rate distribution among the side holes. This can be exploited to determine drainage performances of a cannula using experiments carried out with water rather than blood.

## 5 LIMITATIONS

The comparison between Cases 1 and 2 allowed for an evaluation of the sensitivity of the results to different investigation techniques. Apart from the aforementioned wall effects, there is an uncertainty associated with the use of bi‐dimensional data in such a strong three‐dimensional flow field, in particular regarding the computation of velocity gradients. Using only two velocity components for the stress resulted in an underestimation of shear rate levels of around 50% (Figure 2C). Moreover, flow features such as the counter‐rotating vortex pairs were not captured using the 2D flow field. This study only addressed a lighthouse tip cannula, indicating that drainage performances and other clinically relevant points of interest might differ with multi‐stage or otherwise different geometrical designs. However, some of the fluid structures developing within the cannula are expected to develop for also other cannulae (such as the jets in crossflow developing at the side holes). Moreover, reflection and refraction effects made it challenging to accurately track the flow through the side holes due to the particles being hidden by the light reflected by the walls, especially in the vicinity of the holes, making the validation in these areas complicated.

## 6 CONCLUSIONS

This study identified important flow structures influencing blood behavior in a lighthouse tip drainage cannula, with a pattern similar to jet in crossflow dominating the dynamics of the flow. The most proximal set of holes consistently drained the largest quantity of fluid, and the non‐Newtonian behavior of blood was less relevant in the drainage area. The use of 2D data was found to underestimate the stresses induced within the domain, with the 90° bend of the flow at the side holes being a site of high shear. Such cross‐validated setups could be used to analyze different cannula tip designs.

## AUTHOR CONTRIBUTIONS

Francesco Fiusco: set up the simulations, post‐processed the data and drafted the manuscript. Federico Rorro: performed the experiments. Lars Mikael Broman: designed the concept, read and critically revised the manuscript, suggested appropriate simulation case scenarios with respect to clinical relevance. Lisa Prahl Wittberg: designed the concept, helped with interpretation of experimental and numerical results, read and critically revised the manuscript. All authors reviewed and approved the final manuscript before submission.

## CONFLICT OF INTEREST

The authors declare that they have no conflicts of interest with the contents of this article.

## Supporting information


Video S1

Appendix S1

Appendix S2

Appendix S3
