# Supplementary material for: Numerical and experimental investigation of a lighthouse tip drainage cannula used in extracorporeal membrane oxygenation
Source: Artif Organs. 2022 Oct 21;47(2):330–41. doi: 10.1111/aor.14421 (PMC10092507; doi:10.1111/aor.14421)
Supplement: Supplementary file 2 — Appendix S1 [file AOR-47-330-s002.docx]

# Supplemental Material 1: Meshing & validation

A mesh convergence study was conducted with respect to the convergence of velocity and velocity gradients. Three meshes of 4.3, 9 and 12.6 million cells were considered. The region close to the side holes of the mesh is shown below (a cut plane in the *z*-normal direction).

Various refinements were applied to the mesh in areas of the flow with important dynamics. In the drainage area, a structured-like mesh with a honeycomb structure was prescribed (as shown in Figure S1.1 below), while a refinement was applied on the inner surface of the cannula. All refinements were achieved by prescribing the cell size as a fraction of a *base size*. Among different meshes, the base size was changed, while keeping the reduction factors of the different refinement areas constant (e.g. around the holes the cell size was set as 20% of the base size). In the used medium mesh, the y^+^ on the outer cannula wall was computed to be less than 0.1 over the entire surface, except for a few cells around the proximal holes, where it peaked at 3.

Mesh convergence was assessed by considering velocity and velocity gradients on lines AF, BE, CD as defined in the *Numerical setup* section. Figure S1.2 shows the Frobenius norm of the shear rate and velocity magnitude. The differences between the medium and fine meshes were found negligible in most cases, apart from a slight disagreement in line BE. The difficulty in capturing this region is due to the flow being transitional at this location.

The quality of the mesh was also assessed by considering a metric based on the velocity gradients. In particular, a local flow length scale was devised by considering the velocity magnitude divided by the strain rate modulus. This can be seen as a “velocity gradient length scale”. This value was divided by the cubic root of the cell volume (a measure of the “size” of the cell). Thus, it was possible to compute the number of cells per length scale. A representative snapshot of this quantity is shown in Figure S1.3 for the medium mesh. Note that the color bar is cropped between 0 and 2 to highlight the areas where less than two cells per “unit length” are present. Areas directly close to the walls are treated with wall functions (and moreover, prism layers are grown from the wall, whose length is smaller than the cubic root of the volume. The y^+^ values, as stated above, were less than 1 almost throughout the flow domain.

The use of implicit LES entails stricter requirements for the mesh, as well as for the timestep. In Figure S1.4 is an instantaneous snapshot of the convective Courant number, showing that the CFL condition is fulfilled almost everywhere (and an appropriate number of outer corrector iterations enforces stability).


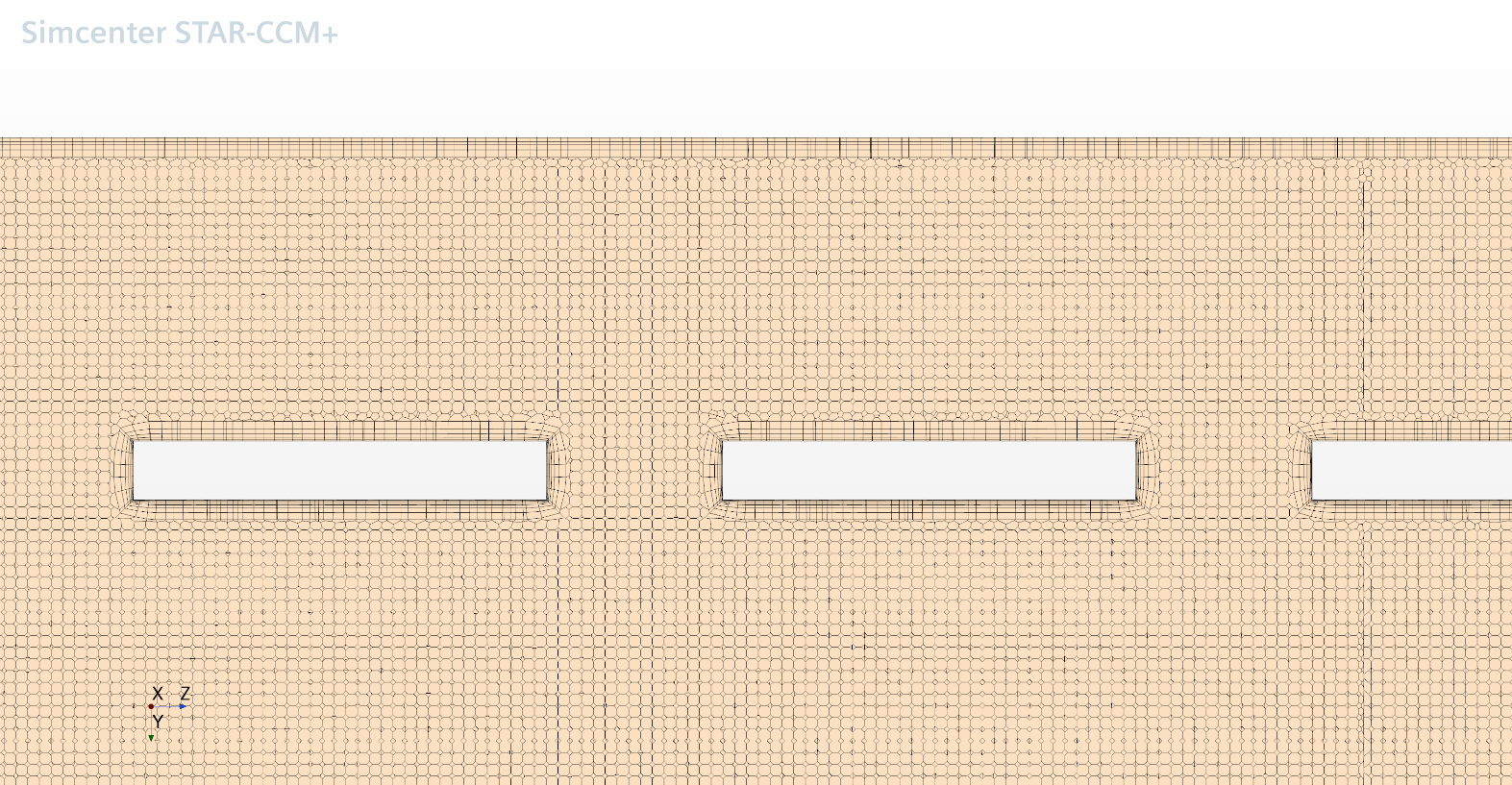


*Figure S1.1: Zoom-in of the computational mesh around the side holes. Seven prism layers were grown from the walls, while a honeycomb-like refinement was applied in the bulk of the drainage area.*
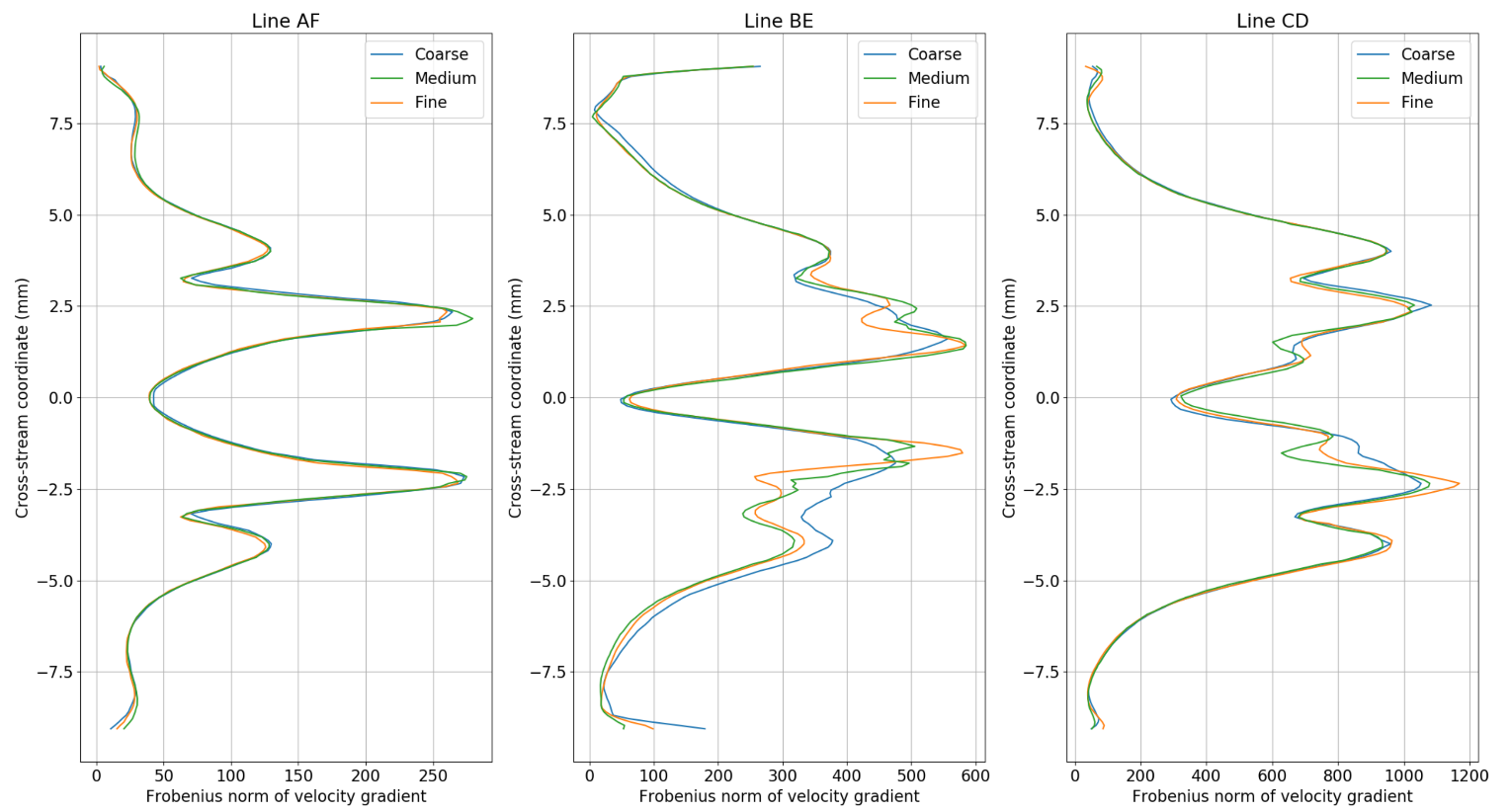


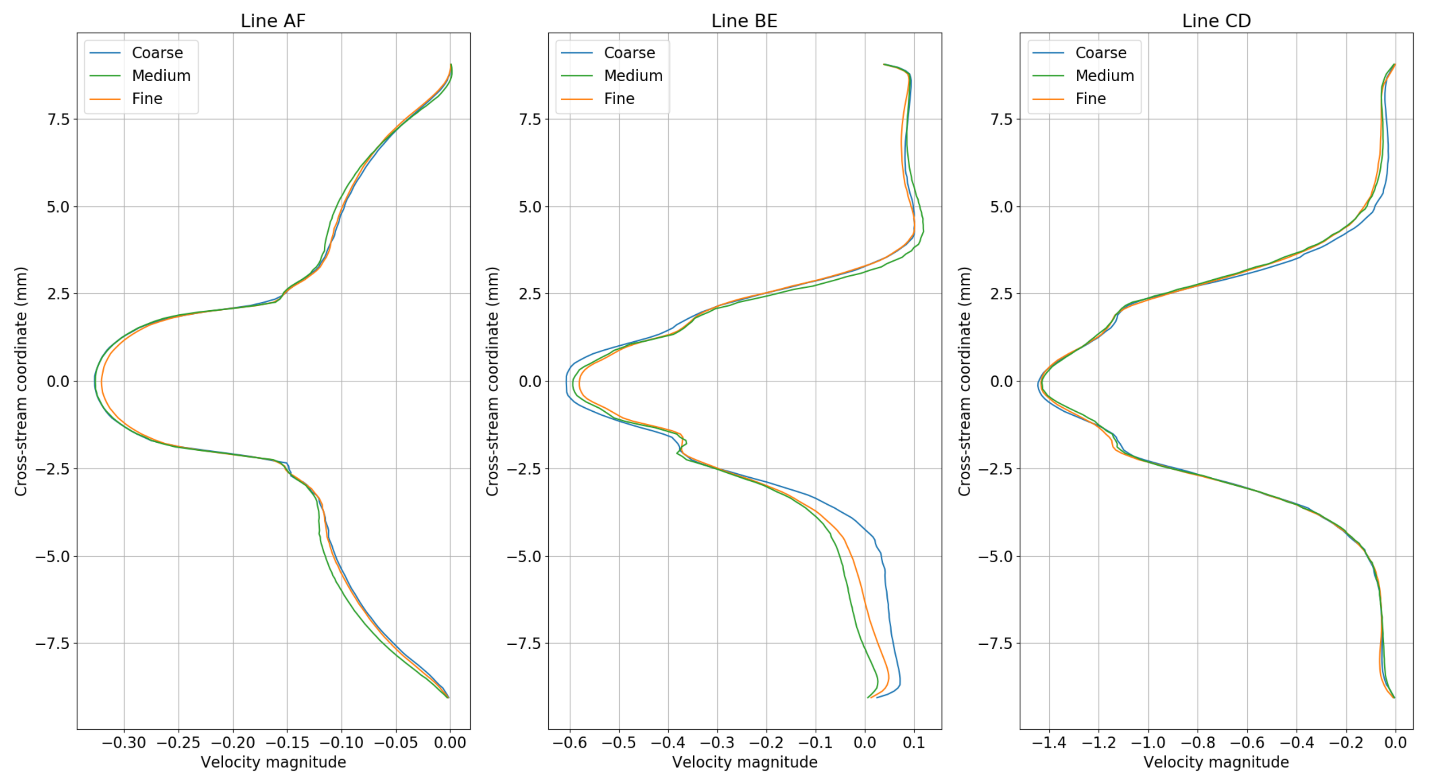


*Figure S1.2: The top panel shows the Frobenius norm of the shear rate tensor on the centerlines of holes AF, BE, CD. In the bottom panel, the velocity magnitude is depicted at the same locations.*


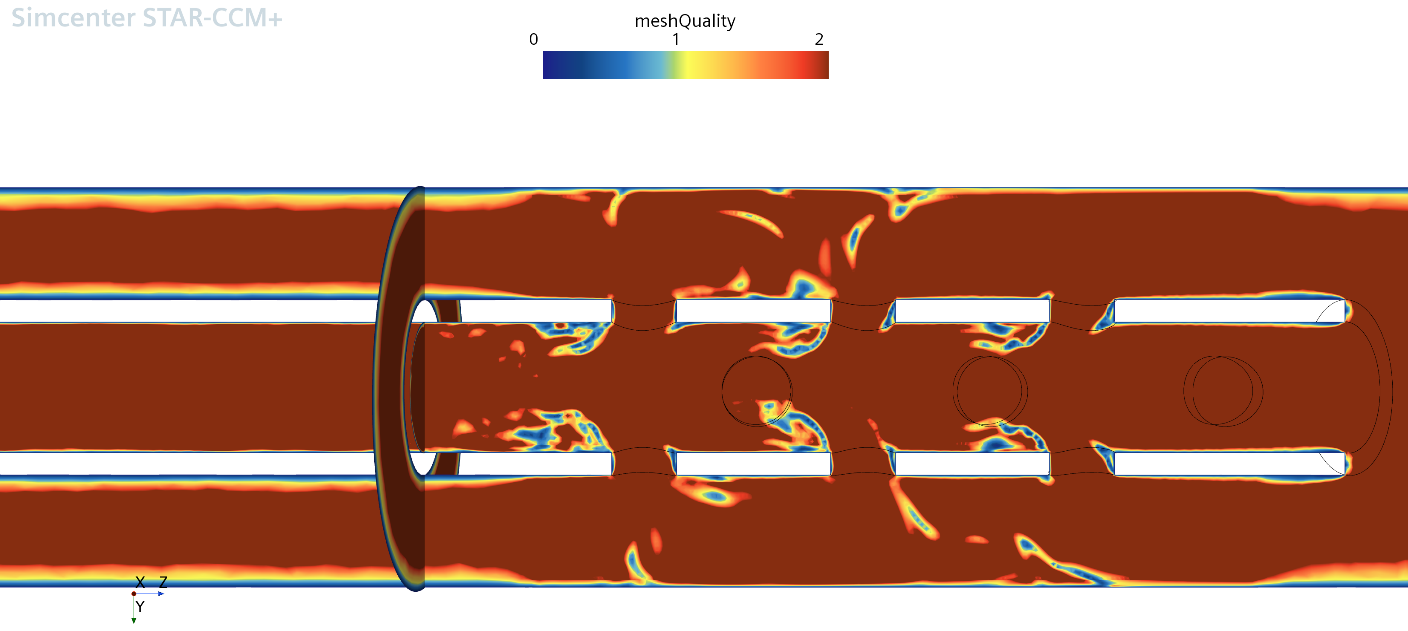


*Figure S1.3: Depiction of the mesh quality parameter interpolated on the cell vertices. Limited regions of low quality are reported in small areas around the walls (since the prism layers have high aspect ratio and their size in the wall-normal direction is much smaller than in the wall-parallel one) and in the recirculation bubbles behind the 90° bends.*


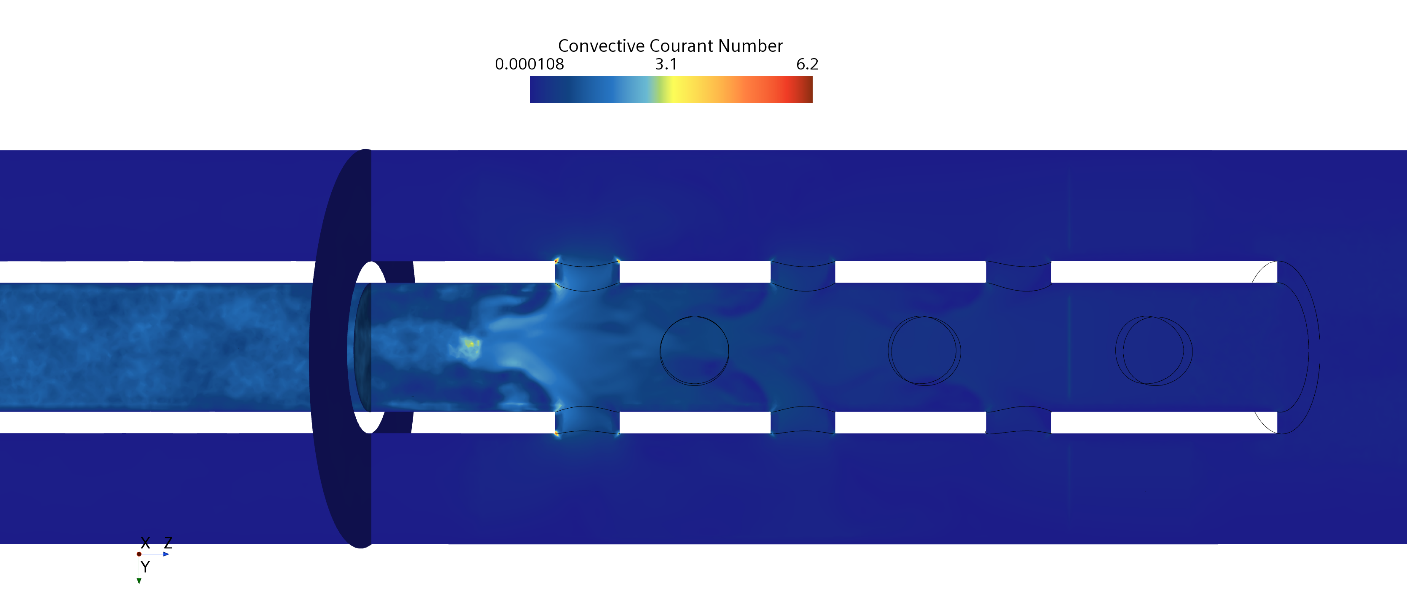


*Figure S1.4: convective Courant number in the drainage area. Its value is much smaller than one almost everywhere.*
