# Supplementary material for: Numerical and experimental investigation of a lighthouse tip drainage cannula used in extracorporeal membrane oxygenation
Source: Artif Organs. 2022 Oct 21;47(2):330–41. doi: 10.1111/aor.14421 (PMC10092507; doi:10.1111/aor.14421)
Supplement: Supplementary file 3 — Appendix S2 [file AOR-47-330-s004.docx]

# Supplementary Material 2: Time-averaged experimental cases

Below are reported the time-averaged streamwise velocity fields for Cases 2 (validation of the baseline numerical Case 1), 7, 8 and 9. The corresponding drainage and vessel flow rates are reported next to the respective Figure.


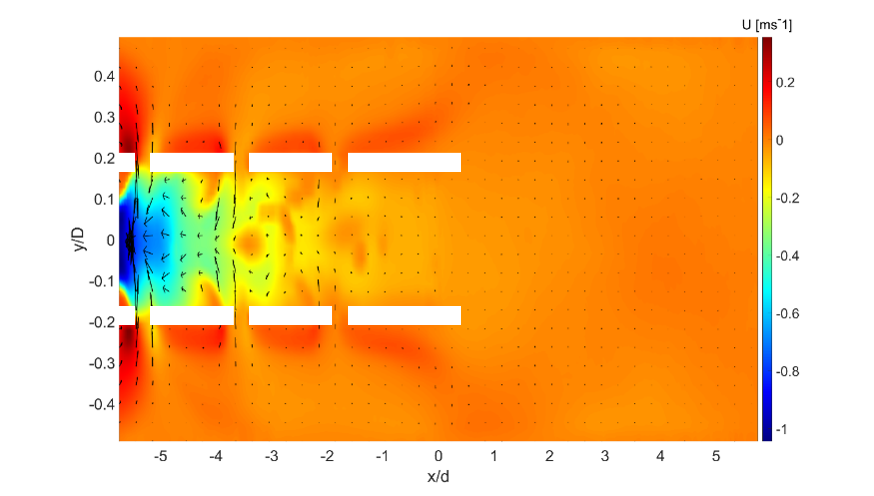


Drainage flow: 2.6 L/min

Co-flow: 1.3 L/min


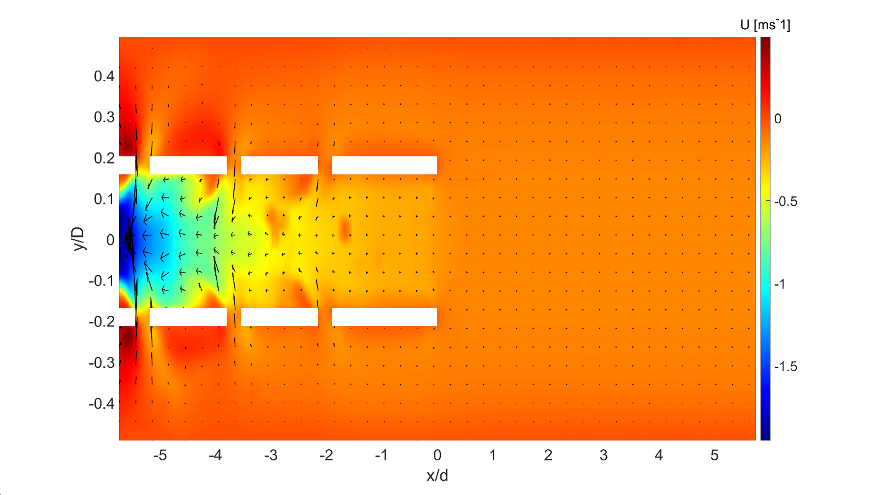


Drainage flow: 1.3 L/min

Co-flow: 1.3 L/min


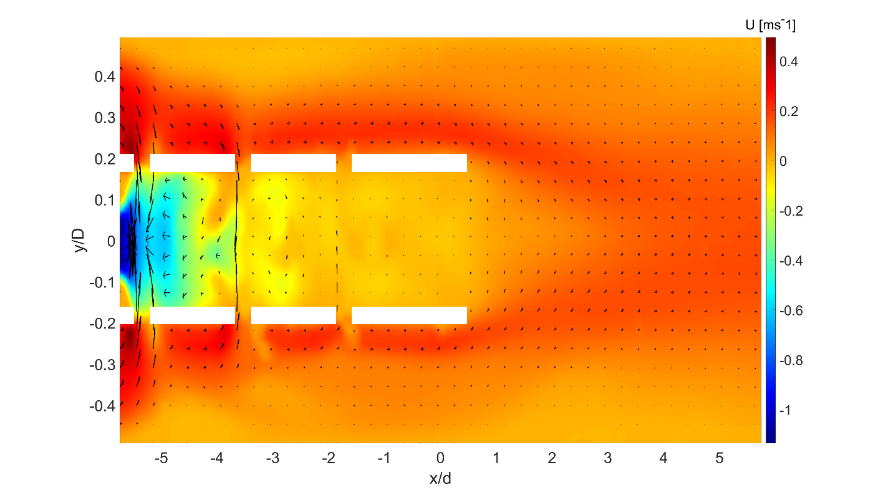


Drainage flow: 1.3 L/min

Co-flow: 2.6 L/min


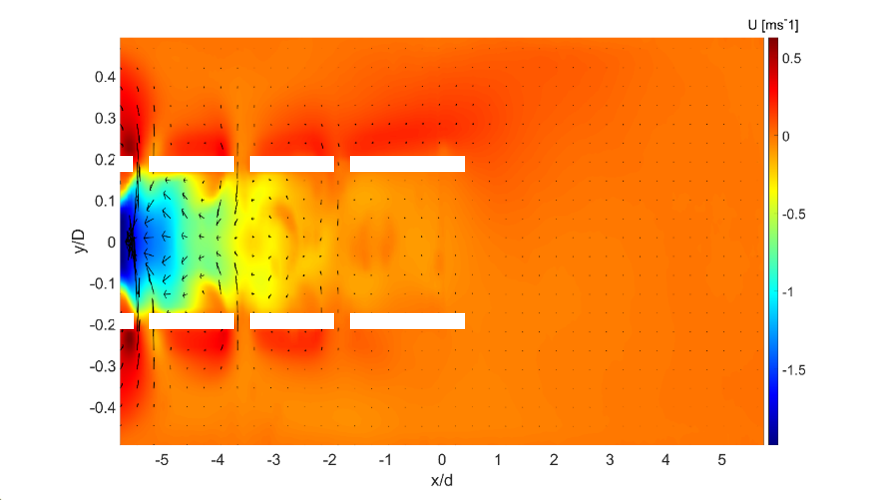


Drainage flow: 2.6 L/min

Co-flow: 2.6 L/min
