# Supplementary material for: Numerical and experimental investigation of a lighthouse tip drainage cannula used in extracorporeal membrane oxygenation
Source: Artif Organs. 2022 Oct 21;47(2):330–41. doi: 10.1111/aor.14421 (PMC10092507; doi:10.1111/aor.14421)
Supplement: Supplementary file 4 — Appendix S3 [file AOR-47-330-s001.docx]

# Supplementary material 4: Shear rate distributions in the experimental cases

In the Figure below are reported the shear rate distributions for the experimental cases 7, 8 and 9.


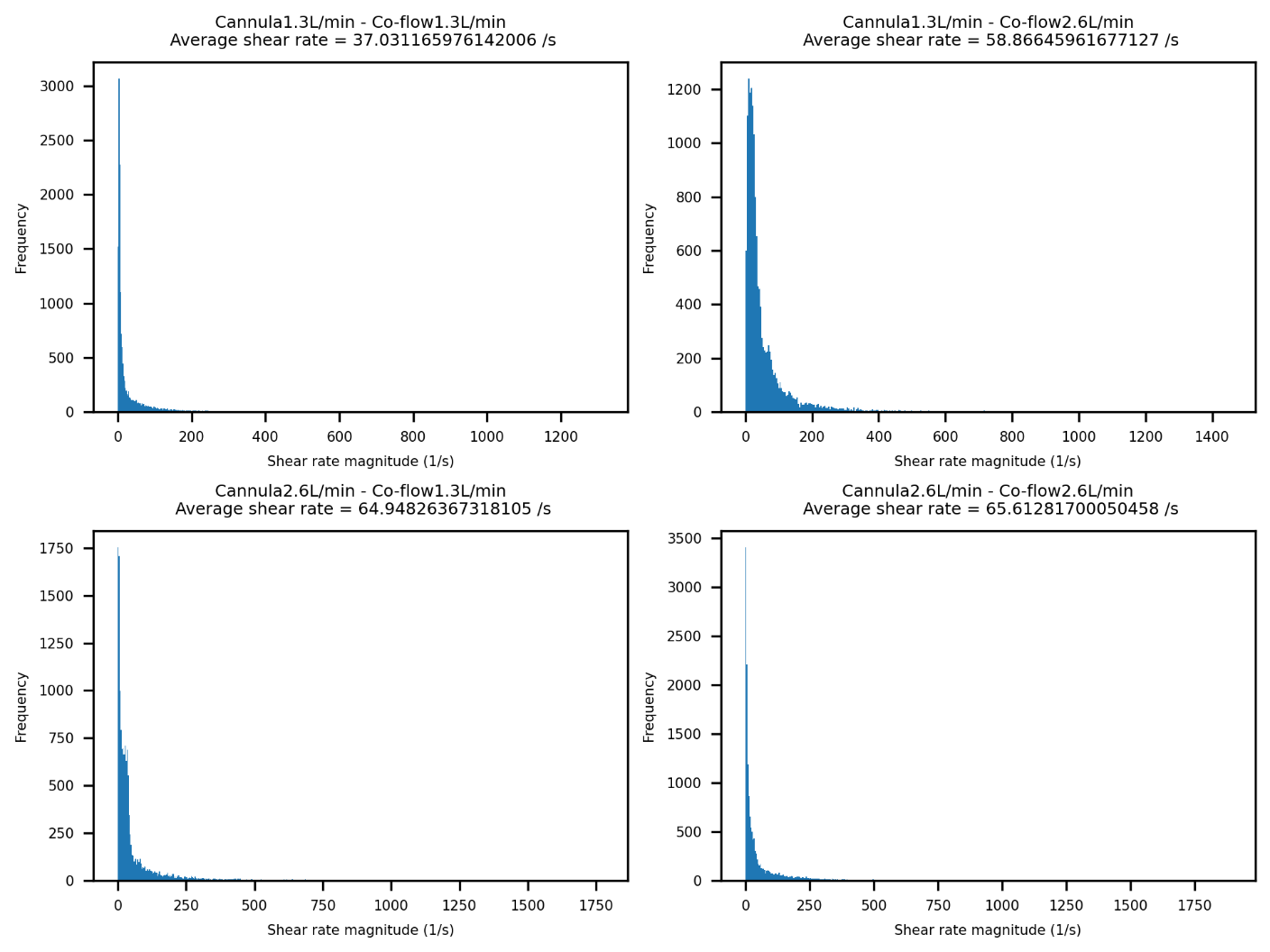


Case 7

Case 8

Case 9

Case 2

Cases 2, 8 and 9 exhibit the highest average shear rate in the domain. Case 8 and 9 show the longest tails, i.e. points in the domain with the highest shear rates.
